# Plasma neurofilament light in Huntington’s disease: A marker for disease onset, but not symptom progression

**DOI:** 10.1016/j.parkreldis.2021.04.017

**Published:** 2021-04-28

**Authors:** Georgia M. Parkin, Jody Corey-Bloom, Chase Snell, Jordan Castleton, Elizabeth A. Thomas

**Affiliations:** a Department of Epidemiology, University of California Irvine, Irvine, CA, USA; b Department of Neurosciences, University of California San Diego, San Diego, CA, USA; c Institute for Interdisciplinary Salivary Bioscience Research, University of California Irvine, Irvine, CA, USA

**Keywords:** Huntington’s disease, Neurofilament light, Plasma, Blood, Biomarker

## Abstract

**Objective:**

To investigate whether plasma NfL levels correlate with clinical symptom severity in premanifest (PM) and manifest HD (HD) individuals, and whether a NfL cut-point could distinguish PM from HD patients with reasonable accuracy.

**Method:**

98 participants (33 control, 26 PM, 39 HD), underwent blood sample collection and clinical assessment, using both UHDRS and non-UHDRS measures, at one academic HD Center. Years to onset (YTO), probability of disease onset in 5 years, and predicted years until 60% onset probability were also calculated. NfL levels were measured using a Meso Scale Discovery assay.

**Results:**

Cohorts differed by age. NfL levels differed significantly across diagnostic groups and were significantly correlated with age. Age-adjusted NfL levels were not correlated with clinical measures in either HD or PM cohorts, but were correlated when cohorts were combined. In PM subjects, NfL levels correlated with YTO, probability of onset in 5 years, and years until 60% onset probability. Using ROC analysis, a NfL cut-point of <53.15 pg/ml distinguished HD from control; <74.84 pg/ml distinguished HD from PM.

**Conclusions:**

These findings implicate plasma NfL as a peripheral prognostic marker for premanifest-HD. Notably, we show that significant correlations between NfL and clinical symptoms are detected only when PM + HD subjects are combined, but not within HD subjects alone. To date, prior studies have investigated the clinical usefulness of NfL exclusively in merged PM + HD cohorts. Our data suggests a biasing of these previous correlations, and hence potentially limited usefulness of plasma NfL in monitoring HD symptom progression, for example, in clinical trials.

## 1. Introduction

Huntington’s Disease (HD) is a progressive, genetic neurodegenerative disorder caused by unstable CAG repeat expansions in the first exon of the Huntingtin gene (*HTT)*. This mutation translates into a polyglutamine repeat in the Huntingtin protein, the length of which varies by CAG expansion number. Pathogenesis in HD arises largely from the expression of the mutant Huntingtin protein (mHtt), leading to the formation of soluble protein oligomers as well as insoluble aggregates that contribute to the disruption of many, predominantly cortical and striatal, intracellular pathways. The expression of mHtt subsequently contributes to the development of a spectrum of clinical signs and symptoms, including chorea, deteriorations in cognition and mood, and changes in personality, ultimately leading to a premature death around 20 years after onset. While the number of CAG repeats has been associated with age of onset and severity, there is still enormous variability in disease onset and progression, even in patients with identical CAG repeat numbers. In fact, it has been estimated that up to 40% of the variation in onset age can be attributed to genes exclusive of *HTT*, with the remaining disparity attributed to environmental factors [1–3].

Due to the autosomal dominant nature of the *HTT* mutation, individuals who carry the mutation will experience symptoms during their lifetime. Symptoms emerge gradually during a premanifest phase, and the precise shift from premanifest to manifest HD may be difficult to anticipate. The potential to predict disease progression and severity during both phases will help individuals and their clinicians best plan for the future ahead. The use of minimally invasive biofluids, and particularly blood biomarkers, have shown diagnostic and prognostic potential in neurodegenerative disorders. Recently, neurofilament light (NfL), the smallest and most abundant subunit in the heteropolymer neurofilament structural protein complex, has emerged as a potential biofluid marker for neurodegenerative disorders, including HD [4–9], due to its exclusively neuronal expression, and release into the extracellular space following axonal degeneration or neuronal damage [10,11].

The aim of this study was to determine whether NfL protein levels correlate with predicted years to onset in premanifest HD and symptom severity in manifest HD, thereby substantiating its usefulness as a prognostic and/or disease activity marker. Through the use of an electrochemiluminescence immunoassay analysis, we have thus compared plasma NfL levels in normal controls, and individuals with premanifest and manifest HD. We have investigated the association between these levels and a battery of clinical measures, as well as currently used prediction methods for disease onset.

## 2. Methods

### 2.1. Human Subjects

This study was approved by the University of California, San Diego (UCSD) Institutional Review Board, in accordance with the requirements of the Code of Federal Regulations on the Protection of Human Subjects. Patients were recruited from the UCSD HDSA Center of Excellence, and carried a diagnosis of HD with family history of the disorder. Premanifest (PM) HD individuals had more than ≥38 CAG repeats, and a Unified Huntington’s Disease Rating Scale (UHDRS) diagnostic confidence rating below 4. Manifest HD patients had a diagnostic confidence rating of 4, indicating that a clinician had ≥99% certainty that the patient presented with “‘unequivocal presence of an otherwise unexplained extrapyramidal movement disorder” [12]. The UHDRS was developed by the Huntington Study Group, and is used as a major outcome measure in controlled clinical trials [12,13]. Normal controls had no reported history of neurological conditions, psychiatric disorders or gout, and no use of psychoactive substances or medications. All participants gave written informed consent prior to sample collection. Demographic and disease data were collected at the time of sample collection, including gender, age, CAG repeat length, years of education, age of onset and family history.

### 2.2. Clinical assessment

PM and HD study participants underwent a single clinical assessment, which included cognitive testing, behavioral and functional measures, and motor ratings. The cognitive battery included the Mini-Mental State Examination (MMSE; score range 0–30) [14], Montreal Cognitive Assessment (MoCA; score range 0–30) [15], Symbol Digit Modalities test (SDMT; score range 0–110) [16] and Stroop word reading test (SWR). Behavioral and psychiatric changes were assessed using the short form Problem Behaviors Assessment (PBA-s; maximum score 160) [17] and the Hospital Anxiety and Depression Scale/Snaith Irritability Scale (HADS-SIS; maximum score 66) [18]. Functional proficiency was evaluated using the UHDRS [12] Total Functional Capacity (TFC; score range 0–13). Motor dysfunction was assessed using the UHDRS Total Motor Score (TMS, score range 1–124). The sum of all maximal chorea sub-scores was also noted (maximum score of 28) [12]. Disease burden was calculated as CAG and Age product (CAP) score [19]. The SWR, SDMT, TFC and TMS were also incorporated into the composite UHDRS score (cUHDRS) as an additional measure of disease progression [20].

Years to onset was calculated as a function of CAG repeat length and parental age of onset, subtracted from the participant’s current age and referred in this study as the ‘Aylward score’ (simple regression equation multiple R = 0.74). This equation was originally formulated in pre-manifest individuals [21]. Probability of disease onset in 5 years, and predicted years to 60% probability of disease onset, were calculated using formulae which incorporate CAG repeat length and current age, and were derived in a premanifest cohort by Langbehn and colleagues [22].

### 2.3. Plasma collection and analysis

Blood from consenting individuals was drawn by venipuncture into 2 ml lavender/EDTA tubes. EDTA/whole blood was mixed well by inversion and spun at 900*g* for 15 min. The supernatant was isolated, aliquoted into 1 ml aliquots, snap frozen and stored at −80 °C. Plasma levels of NfL were measured in duplicate by operators blinded to the clinical state of the participant using Meso Scale Discovery (MSD; Rockville, MD) R-Plex Assay, according to the manufacturer’s instructions. Dilutions in this process included: biotinylated capture antibody, 1:16.5 in MSD Diluent 100 (Cat# R50AA); samples and calibrators, 1:2 in MSD Diluent 12 (Cat#: R50JA); SULFO-TAG^™^ detection antibody, 1:100 in MSD Diluent 11 (CAT# R55BA); and MSD Read Buffer 4X (CAT# R92TC),1:2 in dH2O. The plasma NfL standard curve spanned from 6.1 pg/ml to 12,500 pg/ml, with a manufacturer determined assay lower limit of detection (LLOD) of 5.5 pg/ml. Plasma NfL readout was obtained and concentrations were calculated using the MSD Discovery Workbench version 4.0.12; a calculated concentration was obtained for all plasma NfL values included in this study.

### 2.4. Normalization

Raw data inter-assay variation, calculated through the repeated inclusion of two samples on all plates, was 42–45%. In order to reduce this variation, we determined the average NfL value ratio of samples run on the final assay plate over the values of those same samples when run on previous plates. All sample values on plates other than the final plate run were then multiplied by these average ratios to obtain normalized plasma NfL values. Normalized inter-assay variation was 7–22%.

### 2.5. Statistical analysis

Analyses were conducted with GraphPad Prism version 8.4.2 for Windows (GraphPad Software, La Jolla, CA, USA). Analyses which corrected for age were conducted using IBM® SPSS® Statistics version 25 for Windows (IBM Corp., NY, USA). The ROUT outlier test detected 2 definite outliers (Q = 0.1%) in the NC cohort; these outliers were removed from all analyses. Continuous variables were tested for normality using the Shapiro-Wilks test and analyzed accordingly using parametric and non-parametric tests. Specifically, when comparing cohort characteristics, a one-way analysis of variance (ANOVA) was used to compare age, and the Kruskal-Wallis test was used to compare education level. A Chi-Square test was used to compare gender distribution. The Mann-Whitney test was used to compare CAG repeat number and CAP score between PM and HD cohorts. Plasma NfL levels were compared between cohorts using the Kruskal-Wallis test, and subsequently Quade’s nonparametric one-way rank analysis of covariance (ANCOVA) [23] correcting for age. The Receiver Operating Curve (ROC) analysis was used to determine whether a plasma NfL level cut-point could distinguish manifest HD patients from PM individuals and controls. All correlation analyses between plasma NfL and demographic, disease or clinical data were performed with Spearman’s rho, and subsequently a nonparametric partial correlation test, to correct for age [24]. Statistical significance in these final analyses was considered using the Bonferroni-adjusted p-value cut-off, with the p-value cut-off adjusted by the number of tests in each subheading (demographic, disease or clinical).

## 3. Results

### 3.1. Participant characteristics

Plasma was collected from 98 individuals: 33 normal controls (NC), 26 premanifest *HTT* gene mutation-positive individuals (PM) and 39 manifest HD patients (HD). Demographic characteristics are summarized in Table 1. Cohorts differed significantly by age (Welch’s ANOVA F_2, 57.45_ = 18.55, *p* < .0001); HD and NC cohorts were significantly older than PM (*p* < .0001 and *p*=.0004, respectively). HD patients also had significantly higher CAP scores compared to the PM cohort (U = 13, *p* < .0001).

### 3.2. Diagnostic potential of plasma NFL

**Fig. 1.**
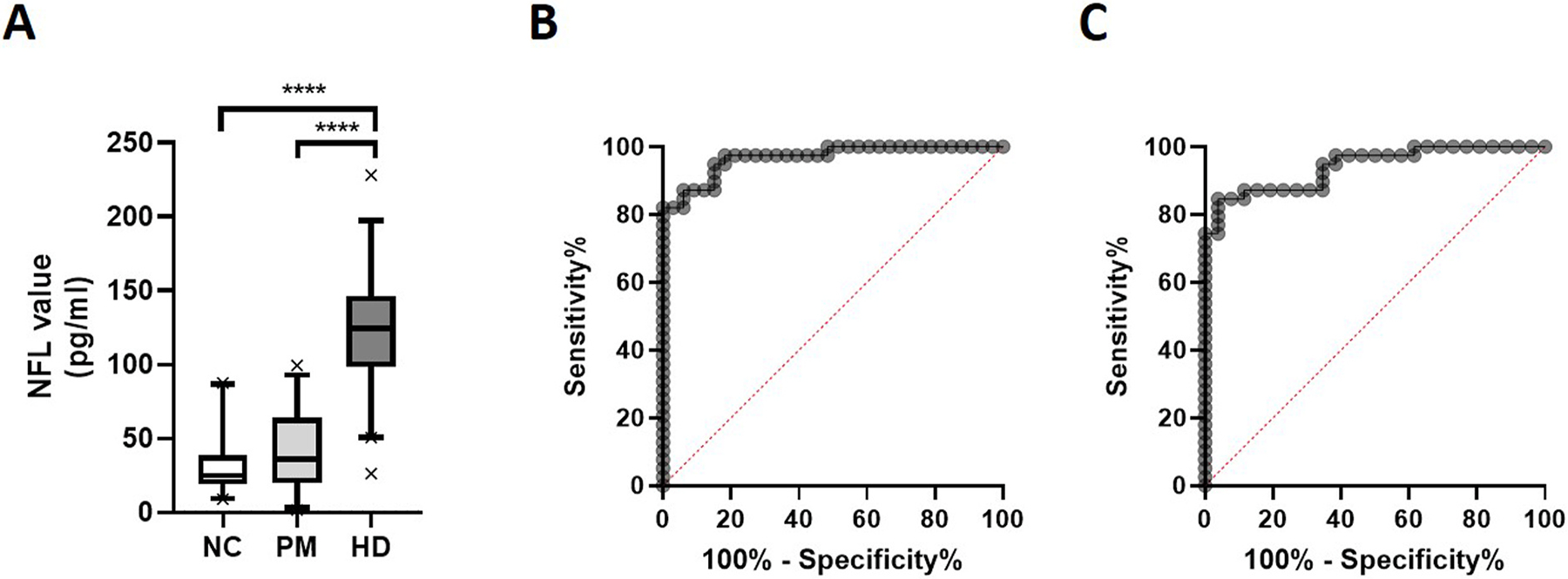
Plasma NfL levels in HD patients, premanifest individuals and normal controls. Plasma NfL levels are significantly increased in manifest HD patients compared to premanifest HD (PM) individuals and normal controls (NC), as determined by Kruskal-Wallis test (p < .0001). Box plots represent median and interquartile range, with whiskers at 5–95 percentiles (A). Receiver Operating Characteristic Curves for plasma NfL in the diagnosis of manifest HD compared to NC (Area Under the Curve (AUC) = 0.92, p < .0001) (B) and PM (AUC = 0.94, p < .0001) (C).

Overall, plasma NfL levels were significantly correlated with participant age (r = 0.50, *p* < .0001) but not gender (U = 949, *p* = .08) or CAG repeat number (r = 0.04, *p* = .72), irrespective of diagnostic cohort. Plasma NfL levels were significantly different across diagnostic groups (Kruskal-Wallis χ^2^ = 58.81, *p* < .0001). Dunn’s posthoc test determined a significant difference between NC (median, interquartile range (IQR): 24.81, 18.94–38.84) and HD (median, IQR: 124.2, 98.24–146.4), and between PM (median, IQR: 35.87, 19.57–63.76) and HD (both adjusted *p*-values < .0001) (Fig. 1A). The ANCOVA significance remained after correcting for age (F_2,97_ = 74.00, *p* < .0001). Subsequent Sheffe’s posthoc test determined significant differences between NC and PM, NC and HD, and PM and HD (all *p*s < .0001).

A ROC analysis determined that a NfL cut-point of <53.15 pg/ml (AUC = 0.97, *p* < .0001) provided a sensitivity of 94.87% and specificity of 84.85% for distinguishing HD patients from controls (Fig. 1B). When comparing PM and HD cohorts, a plasma NfL cut-point of <74.84 pg/ml (AUC = 0.94, *p* < .0.0001) returned a sensitivity of 87.18% and specificity of 88.46% (Fig. 1C).

### 3.3. Correlations between plasma NfL and clinical symptoms in HTT mutation-carrier individuals

**Fig. 2.**
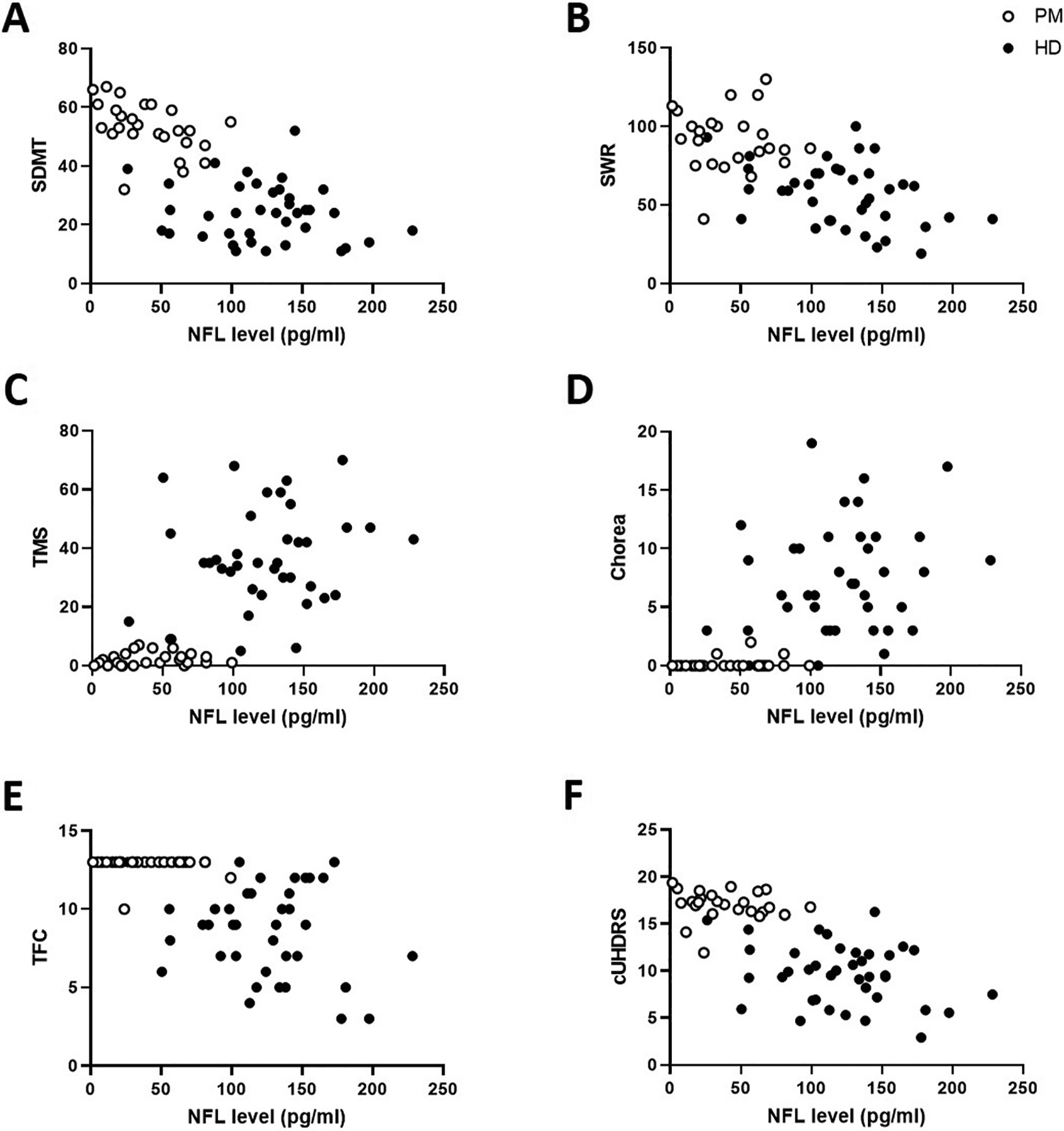
Graphical presentation of clinical scores significantly correlated with plasma NfL in the combined PM + HD cohort. Correlation of plasma NfL levels with Symbol Digit Modality Test score (SDMT; A), Stroop Word Reading score (SWR; B), UHDRS Total Motor Score (TMS; C), UHDRS Chorea score (D), UHDRS Total Functional Capacity score (TFC; E) and composite UHDRS score (cUHDRS; F) in premanifest (PM, open circles) and manifest HD (filled circles) participants. Please refer to Table 2 for Spearman’s rho, Nonparametric Partial Correlation rho, and p values.

All clinical data was analyzed separately in PM and HD cohorts as well as in the combined PM + HD cohort. We found significant correlations between plasma NfL levels and participant age and CAP score in the PM but not the HD cohort. The association between CAP score and NfL was above the Bonferroni-adjusted p-value cut-off score after adjusting for age. NfL levels were correlated with SDMT scores in the PM cohort before, but not after, adjusting for age. We found an association between SWR and NfL levels in the HD cohort that fell just outside the Bonferroni-adjusted p-value cut-off score after adjusting for age. In the PM + HD cohort, MoCA, SDMT, SWR, Chorea, TFC, TMS and cUHDRS were correlated with NfL levels; all measures except the MoCA survived correction for age and multiple comparisons (Table 2). Visual inspection of data suggests that significant correlations in the PM + HD cohort may be driven by a considerable and sudden deterioration in function following symptom onset (see Fig. 2 for graphical presentation of SDMT, SWR, Chorea, TFC, TMS and cUHDRS data).

**Fig. 3.**
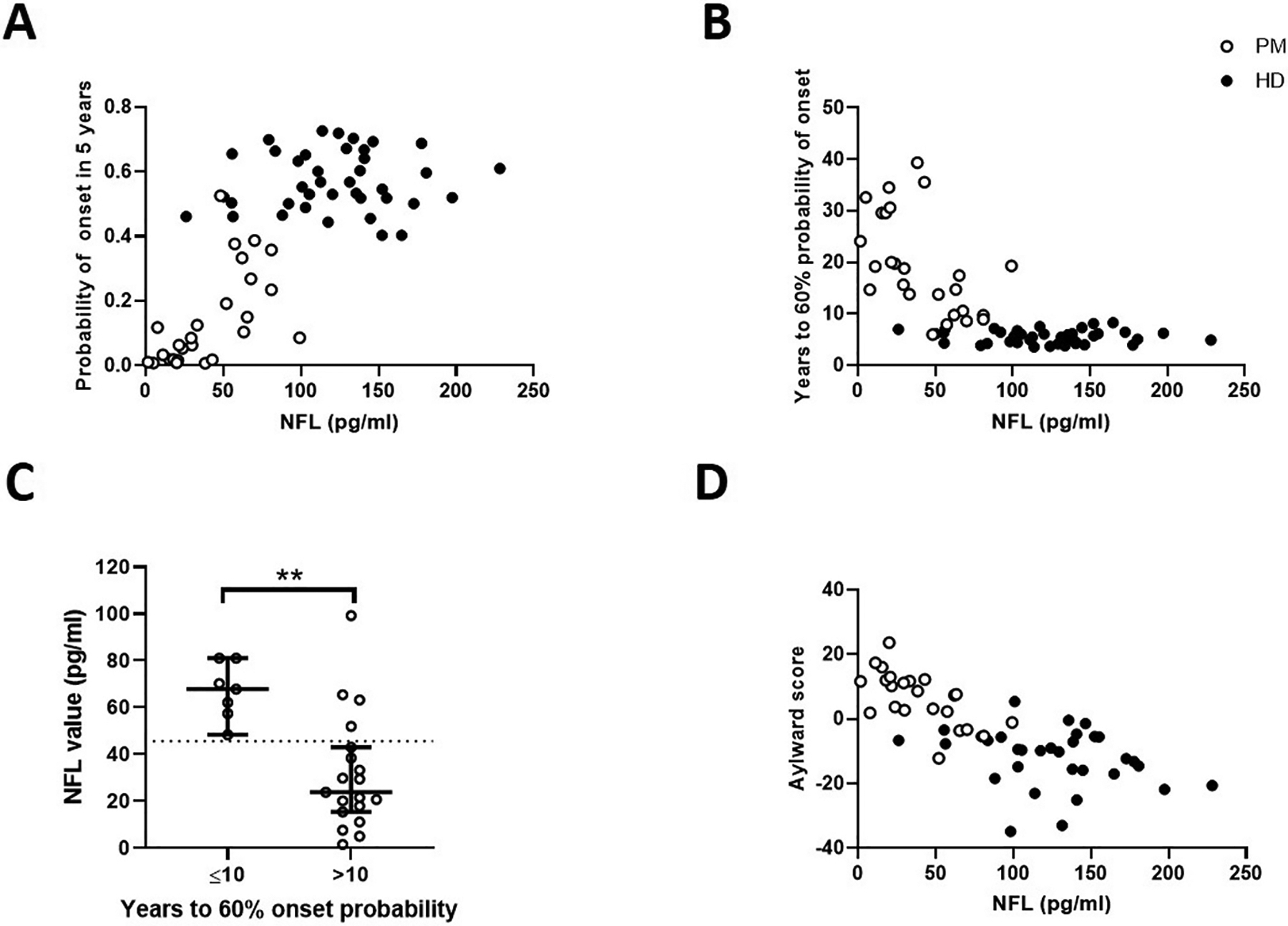
Association between plasma NfL levels and calculations for years to onset. Correlation of plasma NfL levels with Probability of onset in 5 years (A), Years to 60% probability of onset (B, C) and Aylward score (D) in premanifest (PM, open circles) and HD (filled circles) participants. Panel C shows NfL levels in patients who are a predicted ≤10 yrs vs. >10 yrs to onset (post-hoc Mann Whitney *U* test U = 14, p = .001). The dotted line in Figure C represents the optimal NfL value cut-point (45.66 pg/ml) that could distinguish PM individuals with more than 10 years until they reached 60% probability of symptom onset, ≤10 years (AUC: 0.89, sensitivity: 79%, specificity: 100%, p = .002); error bars represent median and 95% confidence intervals. Please refer to text for Spearman’s rho, Nonparametric Partial Correlation rho, and p values for Figures A, B, and D.

Plasma NfL levels were significantly correlated with probability of disease onset in 5 years (rho = 0.71, *p* < .0001; age-adjusted rho = 0.62, *p* = .001) (Fig. 3A, open circles), and predicted years to 60% onset probability (rho = −0.60, *p* = .001; age-adjusted rho = −0.47, *p* = .02) (Fig. 3B, open circles) in the PM, but not the HD, cohort (HD p > .05, Fig. 3A and B, closed circles). An exploratory ROC analysis determined that an NfL cut-point of <45.66 pg/ml (AUC = 0.89, *p* = .002) had a sensitivity of 78.95% and specificity of 100.00% in detecting PM individuals with more than 10 years until 60% disease onset probability (post-hoc Mann-Whitney U = 14, *p* = .001; Fig. 3C). Plasma NfL was also correlated with the Aylward score in the PM cohort, both before (rho = −0.68, *p* < .0001) and after (rho = −0.47, *p* = .03) adjusting for age. The HD cohort did not show a correlation between NfL and Aylward score (Fig. 3D). In the PM + HD cohort, NfL levels correlated with probability of onset in 5 years (rho = 0.70, *p* < .0001; age-adjusted rho = 0.60, *p* < .0001), predicted years to 60% onset probability (rho = −0.69, *p* < .0001; age-adjusted rho = −0.60, *p* < .0001) and Aylward score (rho = −0.75, *p* < .0001; age-adjusted rho = −0.55, *p* < .0001) (Fig. 3A,B,D).

## 4. Discussion

In this study, we found that plasma NfL levels can strongly distinguish manifest HD patients from both PM and control groups, and were significantly correlated with several disease and clinical measures in the PM + HD cohort. These findings mirror those previously reported for CSF and blood NfL levels in HD [4,6–9]. Our findings also validate previously observed significant associations between plasma NfL levels with age [5,10] and CAP score, both in the PM and PM + HD cohort [6]. Importantly, while previously published associations between plasma NfL and clinical data distinguished PM from HD participants graphically, all data had been analyzed using a combined PM + HD cohort [6, 7]. The significant correlations between plasma NfL and SWR, SDMT, Chorea, UHDRS TMS and TFC scores observed in our PM + HD cohort were not present in either PM or HD patients alone, with the possible exception of SDMT in PM and SWR in HD. This suggests that previously reported associations may be driven by the increase in plasma NfL levels from premanifest to manifest HD, and a simultaneous yet not associated decrease in cognitive and motor function. Specifically, and as suggested by the graphical presentation of our data, plasma NfL levels in one cohort may contribute a floor or ceiling effect to an otherwise weak association present in the other, when analyzed together. These results can be compared to those previously reported by Niemelä and colleagues in the CSF of a PM + HD cohort: associations were reported between CSF NfL levels and TFC, TMS, SDMT and all Stroop subscores before correcting for age, whereas only Stroop subscores and TMS remained significant following age correction [25]. The authors did not separate their cohorts into pre- and post-manifest HD, and their data suggests that, similar to our results, these significant correlations may be driven by a debilitating onset of symptoms in the manifest cohort. Likewise, Constantinescu and colleagues found higher levels of CSF NfL in HD patients compared to controls, but no significant associations between CSF NfL and clinical symptoms in manifest HD patients only [8]. However, Vinther-Jensen and colleagues reported an age, CAG and Bonferroni-corrected correlation between CSF NfL and TMS in manifest HD, and an association between NfL and cognitive impairment in their PM + HD cohort, suggesting that NfL levels in CSF might be more reflective of neurodegeneration than plasma NfL [4].

A previously published systematic review of HD clinical measures found that the SDMT was the most sensitive cognitive measure, particularly for premanifest HD, and considered the Stroop test a core assessment component [26]. In our study, the correlation between plasma NfL levels and the SDMT fell within the Bonferroni-adjusted p-value cut-off score in the PM cohort before age adjustment, and the SWR fell just above this score in the HD cohort after age adjustment. While these observations support the potential of plasma NfL as a marker of cognitive decline, it is important to reiterate that neither correlation survived corrections for age. Furthermore, the lack of correlation between plasma NfL and any other clinical measure in either PM or HD cohort individually, suggests limited usefulness of this measure in tracking premanifest or manifest symptom presentation, over and above the use of the clinical measure itself.

Another disease feature that we included in this study was the cUHDRS, a measure of disease progression that incorporates the SWR, SDMT, TFC and TMS [20], and also a primary outcome measure currently used in clinical trials of disease-modifying therapies for HD. Similar to the other clinical symptoms measured in this study, we found that NfL was significantly correlated with the cUHDRS in our combined PM + HD cohort, but not in the HD only subjects. Hence, once again, using NfL to monitor disease symptoms in manifest patients for clinical trials, may have limited value, contrary to what other groups have suggested. Currently, there are several clinical trials using plasma NfL as a biomarker for disease outcome, where it might be expected that anti-HD drugs would result in decreased levels of plasma NfL. However, it is plausible that an anti-HD drug could decelerate neurodegeneration in manifest HD, without a significant impact on clinical symptomatology. Correspondingly, it is possible that current clinical measures are not robust markers of the underlying disease process in manifest HD. These results are likely to have a major impact on the direction and design of future HD clinical trials.

We further add to the current literature by examining the association between plasma NfL levels and three measures of years to disease onset: Aylward score [21], probability of HD onset in 5 years, and years to 60% onset probability [22]. Of these measures, plasma NfL showed the highest correlations with probability of onset in 5 years, and years until 60% onset probability [22], in the PM cohort. These results mirror a previously reported association between CSF NfL levels and probability of onset in 5 years [25].

Associations have previously been reported between either plasma or serum NfL and CSF NfL in a number of neurodegenerative disorders [7, 27–29], and between CSF NfL and CSF mHtt in HD [7,30]. These associations, coupled with our reported correlations between plasma NfL and years to HD disease onset, suggest the presence and progression of subtle disease pathophysiology, discernible by plasma NfL levels and in the absence of symptom presentation. Overall, consideration of both plasma NfL levels and the Langbehn formula [22] when predicting years to onset may, in the future, improve prognostic accuracy. For example, our exploratory analysis suggests that a plasma NfL cut-point of <45.66 pg/ml may distinguish premanifest individuals with >10 years until they reach 60% probability of disease onset. With several potential disease-modifying treatments in development, successful and cost-effective treatment of HD will depend on the ability to target *HTT* mutation-positive individuals who are both premanifest and nearing disease onset. The potential of plasma NfL to complement an estimation of years to disease onset in premanifest individuals is similarly supported by a previous study by Byrne and colleagues, who dichotomized premanifest individuals into early and late premanifest disease, based on median predicted years to disease onset, and found a significant difference in plasma NfL levels between the two groups. A recent study by Scahill and colleagues suggests that CSF NfL has superior diagnostic and prognostic power over plasma NfL in determining years to onset in participants who are an estimated ≥18 years from clinical onset [9]. While the analysis of CSF NfL may be advantageous in such a cohort, the collection and analysis of blood has many practical, financial, and safety-related advantages over the collection of CSF, and our data argues that plasma NfL would be more advantageous in individuals who are nearing symptom onset.

Overall, our data shows that plasma NfL is robustly elevated following HD symptom onset and, further, that plasma NfL may contribute essential prognostic value in premanifest HD with regard to the most appropriate time to initiate disease-modifying therapies. Also, importantly, our data showing a lack of correlation between plasma NfL and manifest HD symptoms, suggests that plasma NfL may have limited value for tracking disease symptoms after disease onset, i.e. in the context of a clinical trial in manifest HD patients.

## Figures and Tables

**Table 1:** Demographic and disease data for subjects used in this study (median, range).

	NC	PM	HD	*p*
**Gender [M/F]**	18/15	11/15	16/23	.47
**Age [years]**	57.0, 25.0–80.0	36.0, 19.0–66.0	57.0, 36.0–86.0	**<.0001**
**Education [years]**	14.5, 12.0–33.0	16.0, 12.0–22.0	15.0, 5.0–21.0	.25
**CAG repeat**	NA	41.0, 38.0–51.0	42.0, 40.0–49.0	.08
**CAP**	NA	299.1,176.2–467.0	513.7, 412.1–839.2	**<.0001**

Statistical tests used: Gender, Chi-square test; Age, One-way ANOVA; Education, Kruskal-Wallis test; CAG repeat and CAP, Mann-Whitney *U* test. Abbreviations: CAP, CAG-Age Product score; F, female; HD, Huntington’s Disease; M, male; NC, normal control; PM, pre-manifest.

**Table 2:** Correlation of plasma NfL levels and clinical measures in *Htt* mutation-carriers.

	PM					HD					PM + HD				
	n	Unadjusted		Age Adjusted		n	Unadjusted		Age Adjusted		n	Unadjusted		Age Adjusted	
		rho	*p*	rho	*p*		rho	*p*	rho	*p*		rho	*p*	rho	*p*
**Demographic data**															
Age	26	.58	**.002**			39	.15	.36			65	.62	**<.0001**		
Education	23	.028	.89	−.04	.87	39	.03	.85	.00	.97	65	−.12	.34	−.15	.23
**Disease data**															
CAG	26	−.13	.53	.27	.20	39	−.07	.66	−.23	.16	65	.12	.34	.50	**<.0001**
CAP	26	.61	**.001**	.46	.02	39	.06	.73	.10	.56	65	.70	**<.0001**	.57	**<.0001**
Age of onset						39	.04	.82	−.23	.16					
PAO	23	.37	.09	.05	.83	32	.02	.92	−.09	.63	55	.07	.64	−.36	.008
**Clinical Data**															
MoCA	26	−.01	.96	−.04	.86	38	−.03	.84	−.09	.61	65	−.42	**<.0001**	−.38	.002
MMSE	26	−.02	.94	.00	>.99	39	.01	.95	−.04	.80	65	−.32	.01	−.31	.01
TUG	26	.01	.96	.07	.76	31	.02	.91	.00	.97	57	.19	.16	.09	.50
SDMT	26	−.56	**.003**	−.44	.03	38	−.11	.48	−.16	.35	64	−.70	**<.0001**	−.58	**<.0001**
SWR	25	−.17	.43	−.21	.33	38	−.37	.02	−.44	.007	64	−.64	**<.0001**	−.63	**<.0001**
PBA-s Total	26	−.17	.40	.04	.85	39	.12	.47	.13	.45	65	.33	.006	.32	.009
HADS-SIS Total	25	−.14	.51	−.02	.93	37	−.01	.95	−.03	.88	62	.12	.34	.04	.73
Chorea	26	.25	.22	.23	.28	39	−.14	.41	.12	.49	65	.69	**<.0001**	.53	**<.0001**
UHDRS TFC	26	−.16	.43	−.16	.46	39	−.16	.35	−.14	.39	65	−.66	**<.0001**	−.52	**<.0001**
UHDRS TMS	26	.30	.14	.16	.46	39	.16	.34	.15	.36	65	.70	**<.0001**	.55	**<.0001**
cUHDRS	26	−.35	.08	−.38	.06	39	−.19	.25	−.21	.21	65	−.70	**<.0001**	−.58	**<.0001**

All analyses conducted using Spearman’s correlation test or a nonparametric partial correlation test. Boldface values survive Bonferroni adjustment for multiple comparisons. CAP, CAG and Age Product score; cUHDRS, composite Huntington’s Disease Rating Scale; HADS-SIS, Hospital Anxiety and Depression Scale- Snaith’s Irritability Scale; MMSE, Mini-mental State Exam; MoCA, Montreal Cognitive Assessment; PBA-s, Problem Behaviors Assessment-short form; SDMT, Symbol Digit Modalities Test; SWR, Stroop Word Reading; TFC, Total Functional Capacity; TMS, Total Motor Score; TUG, Timed Up and Go; PAO, parental age of onset.

## Data Availability

Anonymized summary data will be shared by reasonable formal request from qualified researchers, subject to a data sharing agreement and in compliance with the requirements of the funding bodies and institutions.
